# Dual Specificity Phosphatase 4 Enhances Immunotherapy Response by Inhibiting TGF-β1 Secretion in Hepatocellular Carcinoma

**DOI:** 10.3390/cancers18081289

**Published:** 2026-04-19

**Authors:** Lian-Pan Su, Wei-Yi Wang, Xiao-Dan Ma, Shi-Hui Hao

**Affiliations:** 1 State Key Laboratory of Oncology in South China, Guangdong Provincial Clinical Research Center for Cancer, Sun Yat-sen University Cancer Center, Guangzhou 510060, China; sulp@sysucc.org.cn (L.-P.S.); wangwy3@sysucc.org.cn (W.-Y.W.); 2 Department of Nuclear Medicine, State Key Laboratory of Oncology in South China, Guangdong Provincial Clinical Research Center for Cancer, Sun Yat-sen University Cancer Center, Guangzhou 510060, China

**Keywords:** dual specificity phosphatase 4, hepatocellular carcinoma, immune checkpoint blockade, immune evasion, transforming growth factor-β

## Abstract

**Background**: Tumor immune microenvironment (TIME) heterogeneity limits immunotherapy efficacy in hepatocellular carcinoma (HCC), underscoring the need for predictive biomarkers and therapeutic targets. We previously identified dual specificity phosphatase 4 (DUSP4) as a mediator of sorafenib resistance, but its immunomodulatory role remains unknown. **Methods**: Glypican-3 (GPC3)-specific chimeric antigen receptor (CAR) T-cell cytotoxicity assays were performed to assess the impact of DUSP4 on HCC immune susceptibility. A subcutaneous tumor model using Dusp4-overexpressing cells in female C57BL/6J mice was established to evaluate DUSP4-mediated microenvironment remodeling and anti-PD-L1 therapy efficacy. Bulk RNA sequencing of DUSP4-overexpressing HCC cells identified downstream pathways. Public datasets were interrogated to correlate DUSP4 expression with immune checkpoint blockade (ICB) response and immune infiltration in HCC. **Results**: DUSP4 overexpression significantly enhanced HCC cell susceptibility to CAR-T cell killing in vitro and potentiated anti-PD-L1 efficacy in vivo, accompanied by TIME remodeling. Mechanistically, RNA sequencing revealed DUSP4-mediated downregulation of the TGF-β signaling pathway, functionally confirmed using a neutralizing antibody that abrogated the enhanced CAR-T killing. Public datasets confirmed associations between DUSP4 expression and enhanced immune cytolytic activity with favorable prognostic outcomes in HCC. **Conclusions**: DUSP4 serves as a critical molecular nexus linking targeted therapy resistance to enhanced immunotherapy sensitivity. By attenuating the TGF-β signaling pathway, DUSP4 reprograms TIME toward an immunologically active state, thereby augmenting the efficacy of immunotherapy. These findings establish DUSP4 as a promising dynamic biomarker for guiding sequential therapy in HCC and highlight its potential as a novel therapeutic target to improve outcomes in solid tumor immunotherapy.

## Simple Summary

Hepatocellular carcinoma (HCC) remains a challenging malignancy with limited response to immunotherapy. Although immune checkpoint blockade has shown promising results, its efficacy is often compromised in clinical practice due to the immunosuppressive tumor microenvironment. In this study, we investigated the molecular mechanisms underlying immune resistance and identified DUSP4 as a candidate modulator of this process. Our results demonstrate that elevated DUSP4 levels enhance tumor cell susceptibility to T-cell-mediated killing by suppressing TGF-β1 secretion. Importantly, upregulated DUSP4 expression significantly improves immunotherapy efficacy in both mouse models and patient data analyses. These findings reveal a previously unrecognized mechanism regulating the tumor immune microenvironment and suggest that targeting the DUSP4/TGF-β1 axis could improve outcomes for patients with HCC.

## 1. Introduction

Hepatocellular carcinoma (HCC) ranks as the sixth most prevalent and third most lethal malignancy globally [1,2,3]. Although diverse therapeutic strategies have been developed, the survival benefits for HCC patients remain unsatisfactory, characterized by high rates of recurrence and resistance to conventional therapies [4]. Recent advances in immunotherapy, such as immune checkpoint blockade (ICB) therapy, cancer vaccines, adoptive cell therapy (ACT), and combination therapies, have shown promise in enhancing anti-tumor responses [5,6].

ICB therapy primarily targets inhibitory receptors such as PD-1, CTLA-4 and LAG-3, functioning by transmitting inhibitory signals of T cells [7,8]. Although the upregulation of immune checkpoint molecules represents one of the key mechanisms of immune escape in HCC, the clinical efficacy of anti-PD-1/PD-L1 monotherapy remains limited, with objective response rate (ORR) observed in only 15–20% of patients [5]. This constrained therapeutic effect may be attributed to the multifactorial character of immune evasion in HCC, which includes loss or mutation of tumor antigens, infiltration of immunosuppressive cells, and overexpression of immunosuppressive cytokines [7,9,10]. Inflammatory mediators critically influence the tumor immune microenvironment (TIME) and drive HCC progression [9]. Understanding immune evasion mechanisms within the tumor microenvironment (TME) is key to improving immunotherapy outcomes in HCC.

Transforming growth factor-beta 1 (TGF-β1) is a pivotal regulator in HCC [11,12,13]. In early hepatocarcinogenesis, it primarily functions as a tumor suppressor by inducing cell cycle arrest and apoptosis [14]. In contrast, in advanced disease, TGF-β1 switches to a potent tumor promoter, driving epithelial–mesenchymal transition (EMT), fostering a suppressive TIME, and reprogramming the tumor stroma [13,14]. This functional pleiotropy makes TGF-β1 a compelling but challenging therapeutic target. While TGF-β pathway activity can stratify HCC patients and predict outcomes [15,16], the clinical translation of TGF-β1-targeted therapies remains difficult. This is largely due to its indispensable roles in tissue homeostasis and a limited understanding of the upstream mechanisms that precisely control its activation, latency, and cellular specificity.

The dual specificity phosphatases (DUSPs) comprise a family of stress-induced enzymes that exert feedback inhibition on the mitogen-activated protein kinase (MAPK) signaling pathway and play crucial roles in tumor initiation, malignant progression, therapy resistance, and the interaction between tumor cells and immune cells [17]. DUSPs may play different roles in different tumors. DUSP9 is highly expressed in head and neck squamous cell carcinoma tissues and exhibits a negative correlation with the level of immune cell infiltration [18]. Conversely, in the lung tumors of EGFR^del^/DUSP22 knockout mice, the number of CD8^+^ T cells and NKT cells is diminished, and the level of IFN-γ within the tumor is reduced [19]. Our previous findings have mainly focused on tumor-intrinsic aspects, introducing DUSP4 as a negative regulator of sorafenib-induced ferroptosis [20], but its impact on HCC TIME has not been clarified.

This study demonstrates that genetic overexpression of DUSP4 in HCC cells enhances sensitivity to T-cell therapy and remodels the suppressive TIME by suppressing TGF-β1. Our findings elucidate a previously unrecognized mechanism of immune evasion in HCC and underscore the therapeutic potential of targeting DUSP4. Consequently, this work provides a foundational framework for developing novel and more effective immunotherapeutic strategies against HCC.

## 2. Materials and Methods

### 2.1. Data Acquisition and Analysis

R v4.3.0 was used for computational analyses. GEO datasets GSE202069 (41 HCC, anti-PD-1), GSE235863 (15 HCC, anti-PD-1+lenvatinib), and GSE140901 (24 HCC, anti-PD-1/PD-L1) were analyzed. Differentially expressed genes (DEGs) between immunotherapy responders and non-responders were defined by |log_2_FC| ≥ 1 and FDR < 0.05. ICB response was assessed by TIDE [21] and predictive value of DUSP4 by receiver operating characteristic (ROC), with high/low groups defined by dataset-specific median DUSP4 expression.

For the TCGA-LIHC (liver hepatocellular carcinoma) cohort (*n* = 363), the following analyses were performed. Immune scores were calculated using the ESTIMATE package [22]. The Immunophenoscope (IPS) algorithm in the IOBR package was applied to compute scores for MHC molecules, immune checkpoints, effector cells, and suppressor cells [23]. Briefly, the MHC molecule score, which reflects tumor antigen processing and presentation, was estimated based on genes including HLA-A, HLA-B, HLA-C, B2M, HLA-DRA, and HLA-DRB1; the immune checkpoint score, representing T-cell activation and tolerance, was computed based on a panel of genes such as PDCD1, CD274, CTLA4, LAG3, HAVCR2, and TIGIT; the effector cell score, indicative of direct tumor killing activity, was derived from representative genes including CD8A, GZMB, PRF1, IFNG, TBX21, and KLRK1; and the suppressor cell score, reflecting the ability to inhibit anti-tumor immune responses, was estimated using key genes such as FOXP3, IL2RA, CD163, TGFB1, IL10, and CD33.

The MCPcounter algorithm in the IOBR package was applied to assess DUSP4–immune infiltration associations [24]. Overall survival (OS) and progression-free survival (PFS) in pan-cancer immunotherapy patients were evaluated using KM Plotter (https://kmplot.com/analysis/) (accessed on 27 July 2025).

### 2.2. Cell Culture

The cell lines Huh-7 (RRID: CVCL_0336), Hep3B (RRID: CVCL_0326), HEK293T (RRID: CVCL_0063), and Hepa1-6 (RRID: CVCL_0327) were procured from the American Type Culture Collection. Cell lines were authenticated by short tandem repeat profiling and routinely tested for Mycoplasma contamination. All cells were maintained in DMEM (Invitrogen, Carlsbad, CA, USA) containing 10% fetal bovine serum (FBS) (PAN, Aidenbach, Germany), 1% penicillin/streptomycin. All cells were incubated at 37 °C in a humidified atmosphere with 5% CO_2_.

### 2.3. Animal Models

Female C57BL/6J (RRID: IMSR_JAX: 000664) mice, aged 6–8 weeks and weighing 18–22 g, were obtained from Beijing Vital River Laboratory Animal Technology (Beijing, China). All mice were maintained under specific-pathogen-free conditions and were immunocompetent. Animals were acclimatized for one week prior to the start of experiments. For the Hepa1-6 models, 1 × 10^6^/100 µL cells were subcutaneously injected into the right flank of C57BL/6J mice. Mice (*n* = 24) were then randomly assigned to four groups (*n* = 6/group), with the individual mouse serving as the experimental unit. To evaluate the effect of immunotherapy on tumor growth, mice received anti-PD-1 Ab (5 mg/kg, every other day) or PBS after tumor implantation for 7 days. Tumor size was routinely monitored every 4 days. Tumor volumes were calculated by the following formula: tumor volume = 0.5  ×  length  ×  width^2^. All animal procedures were performed under isoflurane anesthesia (2–3% inhalation) during tumor cell inoculation and tumor size measurement to minimize pain and distress. No mortality or severe morbidity occurred in any group during the study. Mice were euthanized at the specified time points, and tumors were excised for flow cytometry analysis.

### 2.4. Plasmid Construction and Transfection

The full-length DUSP4 was cloned into the pHAGE lentiviral vector expressing the surface marker mThy1.1 or GFP. The sgRNA targeting DUSP4 or non-targeting gRNAs was cloned into the pXPR lentiviral vector expressing mTHY1.1. Lentiviral particles were generated in HEK293T cells. Briefly, HEK293T cells were seeded into a T175 flask one day before transfection in DMEM containing 10% FBS to reach 70% confluence on the day of transfection. On the day of transfection, Lenti-vector and an empty vector control were co-transfected with psPAX2 (RRID: Addgene_12260) and pMD2.G (RRID: Addgene_12259). The transfection medium was removed after 6 h and replaced with DMEM supplemented with 20% FBS. The lentivirus supernatant was collected at 48 h and 72 h post-transfection. Following ultracentrifugation for concentration (20,000 rpm at 4 °C for 90 min), the lentiviral particles were resuspended in cold sterile 1 × HBSS with 5% sucrose and stored at −80 °C. The sgRNA details are in Table S1.

Tumor cells were cultured to a cell density of 50–70%, after which a mixture of lentiviral particles (MOI = 3) and Polybrene (8 µg/mL) was added to the tumor culture medium. Twenty-four hours post-infection, the medium was replaced with fresh medium. Three to five days post-infection, positive cells were sorted by fluorescence-activated cell sorting and then continued to culture.

### 2.5. Western Blotting

Cells were rinsed with PBS and then lysed on ice with RIPA lysis buffer (Thermo Fisher Scientific # 89900, Waltham, MA, USA) containing Protease Inhibitor Cocktail (MCE # HY-K0010, Monmouth Junction, NJ, USA) for 15 min. The lysates were then cleared by centrifugation at 15,000 rpm at 4 °C. Protein concentrations were measured using the BCA protein assay (Thermo Fisher Scientific # 23228, Waltham, MA, USA), adjusted to equal levels across samples, and then the samples were boiled at 95 °C for 5 min after the addition of SDS loading buffer (5×). Equal amounts of protein samples were subjected to SDS-PAGE and transferred to a PVDF membrane (Millipore # ISEQ00010, Darmstadt, Germany). The membranes were blocked with 5% skim milk at room temperature for 1 h and then incubated with primary antibodies overnight at 4 °C with gentle shaking. The following day, the membranes were washed with TBST (10 mM Tris-HCl, pH 8.0, 150 mM NaCl, 0.1% (*v*/*v*) Tween 20) and incubated with secondary HRP-conjugated antibodies in TBST at room temperature for 1 h. Finally, ECL (Millipore # WBKLS0500, Darmstadt, Germany) was applied for signal detection. The antibody details are in Table S2.

### 2.6. Quantitative Real-Time Polymerase Chain Reaction (qRT-PCR)

Total RNA was extracted from cells using Trizol reagent (Invitrogen # 15596018, Carlsbad, CA, USA) and reverse transcribed using the HiScript III 1st Strand cDNA Synthesis Kit (Vazyme # R312-01, Nanjing, China) according to the manufacturer’s protocol. qRT-PCR was carried out with the SYBR Green Master Mix (Vazyme # Q712-02, Nanjing, China) on a CFX96 Touch System (Bio-Rad, Hercules, CA, USA). GAPDH was used as an internal control. The primers are listed in Table S3.

### 2.7. Immunohistochemistry (IHC)

The paraffin tissues were hydrated and incubated with 0.3% hydrogen peroxide for 15 min at room temperature, followed by high-pressure antigen retrieval in EDTA buffer (PH 8.0). The sections were blocked in 1:50 goat serum for 1 h at room temperature and incubated with primary antibody at 4 °C overnight. Universal secondary antibody (ZSGB-Bio # PV-6000-18, Beijing, China) was incubated on the slide for 1 h at room temperature. Immunoreactivity was detected using a DAB reagent kit (ZSGB-Bio # ZLI-9017, Beijing, China). The antibody details are in Table S2.

### 2.8. T-Cell Culture and Transduction

Peripheral blood mononuclear cells (PBMCs) from healthy donors (Guangzhou Bairuikang Biotechnology #AP-010D, Guangzhou, China) were revived and cultured for one day in X-VIVO-15 medium (Lonza #02-060Q, Basel, Switzerland) without cytokines. T cells were activated using anti-human CD3 (BioLegend #317326; RRID: AB_11150592, San Diego, CA, USA) and anti-human CD28 (BD #555725; RRID: AB_396068, Franklin Lakes, NJ, USA) antibodies for 24 h. T cells were cultured and expanded in X-VIVO-15 medium (Lonza #02-060Q, Basel, Switzerland) supplemented with 10% FBS and 20 ng/mL recombinant human IL-2 (BioLegend #575408, San Diego, CA, USA). The glypican-3 (GPC3)-specific CAR was transduced into T cells after one day of activation.

### 2.9. Tumor Cell and T-Cell Co-Culture Experiments

For human T-cell co-culture assay, activated GPC3-specific CAR-T cells were added at the indicated effector-to-target (E: T) ratios. After 24 h of co-culture, T cells were removed and stained with indicated antibodies.

### 2.10. Analysis of Tumor-Infiltrating Lymphocytes

Tumors were minced into 1–3 mm^3^ fragments and dissociated using the GentleMACS dissociator in a solution containing 1 mg/mL collagenase type IV (Sigma-Aldrich #C5138, St. Louis, MO, USA), 20 U/mL DNase type IV (Sigma-Aldrich #D5205, St. Louis, MO, USA), and 0.1 mg/mL hyaluronidase type V (Sigma-Aldrich #H6254, St. Louis, MO, USA) for 30 min at 37 °C. The resulting cell suspension was passed through a 70 μm filter to obtain single cells, with a small fraction used for flow cytometry.

### 2.11. Flow Cytometry

Flow samples were stained using 1 × 10^6^ cells. Compensations and voltages were adjusted using single-color controls. For intracellular staining of cytokines, T cells were stimulated with tumor cells for 12 h. Then, 10 μg/mL Brefeldin A (MCE # HY-16592-100mg, NJ, USA) was added into medium 6 h before harvesting. The harvested cells were subsequently fixed, permeabilized, and stained with indicated antibodies. Samples were analyzed on a Fortessa flow cytometer (BD, Franklin Lakes, NJ, USA), and data were analyzed by flowJo software v10.8.1. The antibody details are in Table S2.

### 2.12. Patient Cohort

Our research centered on a group of 44 patients with HCC who had previously undergone curative liver resection at the Sun Yat-sen University Cancer Center in China. The criteria for patient inclusion encompassed: (1) adult patients with HCC who had previously undergone radical hepatectomy; (2) a confirmed pathological diagnosis of HCC; (3) an exclusion of any prior exposure to chemotherapy, immunotherapy, or radiotherapy before surgery; and (4) documented administration of sorafenib treatment following tumor recurrence. The ethical underpinning of our study was established through official approval by the Institutional Review Board of Sun Yat-sen University.

### 2.13. Statistical Analysis

Data are presented as mean ± standard deviation (SD). Normality was assessed using the Shapiro-Wilk test, and homogeneity of variances was evaluated using Levene’s test. For comparisons between two groups, unpaired Student’s *t*-test was used. For comparisons among multiple groups, one-way or two-way analysis of variance (ANOVA) was used, followed by Tukey’s post hoc test for multiple comparisons when appropriate. The in vivo experimental unit was the individual mouse, and statistical analyses were performed with *n* representing the number of mice per group. Statistical analysis was performed using GraphPad Prism (version 9.0, RRID:SCR_002798). A *p* value of less than 0.05 was defined as significant.

## 3. Results

### 3.1. DUSP4 Shapes an Immunologically Active TIME to Potentiate ICB Efficacy in HCC

**Figure 1:**
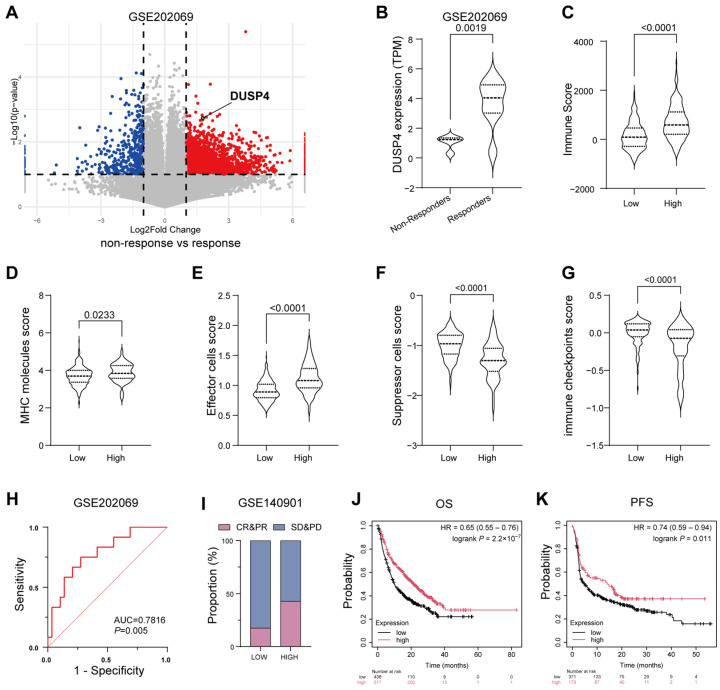
The relationship between DUSP4 expression and resistance to immune checkpoint blockade (ICB) therapy. (**A**) Volcano plot showing the DUSP4 expression was upregulated in responder group (GSE202069). (**B**) Violin Plot showing the difference in DUSP4 expression levels between the non-responder and responder group (GSE202069). Dashed lines within violin denote the median (center) and the upper/lower quartiles (others). (**C**) Association of DUSP4 expression levels (high/low) with tumor immune scores in TCGA-LIHC cohort, as quantified by the ESTIMATE package (*n* = 363). Dashed lines within violin denote the median (center) and the upper/lower quartiles (others). (**D**–**G**) Association of DUSP4 expression levels (high/low) with tumor IPS score in TCGA-LIHC cohort, as calculated by the IPS algorithm from the IOBR package (*n* = 363). Dashed lines within violins denote the median (center) and the upper/lower quartiles (others). (**H**) ROC curve showing the predictive efficacy of DUSP4 levels in hepatocellular carcinoma (HCC) for ICB therapy response (GSE202069). (**I**) Bar chart showing the DUSP4 expression levels in complete response (CR)/partial response (PR) vs. stable disease (SD)/progressive disease (PD) patients (GSE140901). (**J**,**K**) Overall Survival (OS) and progression-free survival (PFS) of pan-cancer patients with high DUSP4 expression level and low DUSP4 expression level following ICB therapy.

To define the role of DUSP4 in shaping the TIME of HCC, we first analyzed transcriptomic data from the GSE202069 cohort [25], which included tumor tissues from 17 HCC patients treated with anti-PD-1 monotherapy. Our analysis revealed that responders to ICB exhibited significantly higher tumor DUSP4 expression compared to non-responders (Figure 1A,B). This finding was further validated in an independent cohort (GSE235863) (Figure S1A,B). To further characterize the immunomodulatory role of DUSP4, ESTIMATE analysis was performed on TCGA-LIHC dataset. The immune score generated by this algorithm provides a robust measure of global immune cell infiltration in tumors; higher scores reflect an immune-enriched “hot” tumor microenvironment, which is usually associated with improved sensitivity to immunotherapy. Our analysis demonstrated that DUSP4-high HCCs had markedly higher immune scores, implying increased immune cell infiltration (Figure 1C). Consistent with this, IPS algorithm analysis of TCGA-LIHC dataset showed that DUSP4 expression significantly affected all four IPS components. DUSP4-high tumors are characterized by elevated MHC molecule and effector cell scores, coupled with reduced immune checkpoint and suppressor cell scores, which collectively reflect a tumor micro-environment more favorable to ICB response (Figure 1D–G).

Receiver operating characteristic (ROC) curve analysis of the GSE202069 cohort further demonstrated that DUSP4 expression exhibited strong predictive power for immunotherapy response (AUC = 0.7816) (Figure 1H). Consistently, in an independent cohort of 24 HCC patients receiving ICB therapy (GSE140901), individuals with high DUSP4 expression displayed a significantly higher objective response rate (complete response plus partial response) (Figure 1I). Extending our analysis to a pan-cancer context, TCGA data indicated that high DUSP4 expression was associated with improved survival following ICB therapy across multiple cancer types (Figure 1J,K).

In summary, our integrated multicohort analyses demonstrate that DUSP4 expression is linked to an immunologically active TIME and serves as a potential biomarker for predicting enhanced ICB efficacy in HCC.

### 3.2. DUSP4 Potentiates the Anti-Tumor Efficacy of T Cells In Vitro

To investigate the functional role of DUSP4 in modulating T-cell-mediated cytotoxicity, we established DUSP4-knockout and DUSP4-overexpressing cell lines in Huh-7 and Hep3B cells, with the efficiency of manipulation confirmed by Western blot (Figure S2A,B).

**Figure 2:**
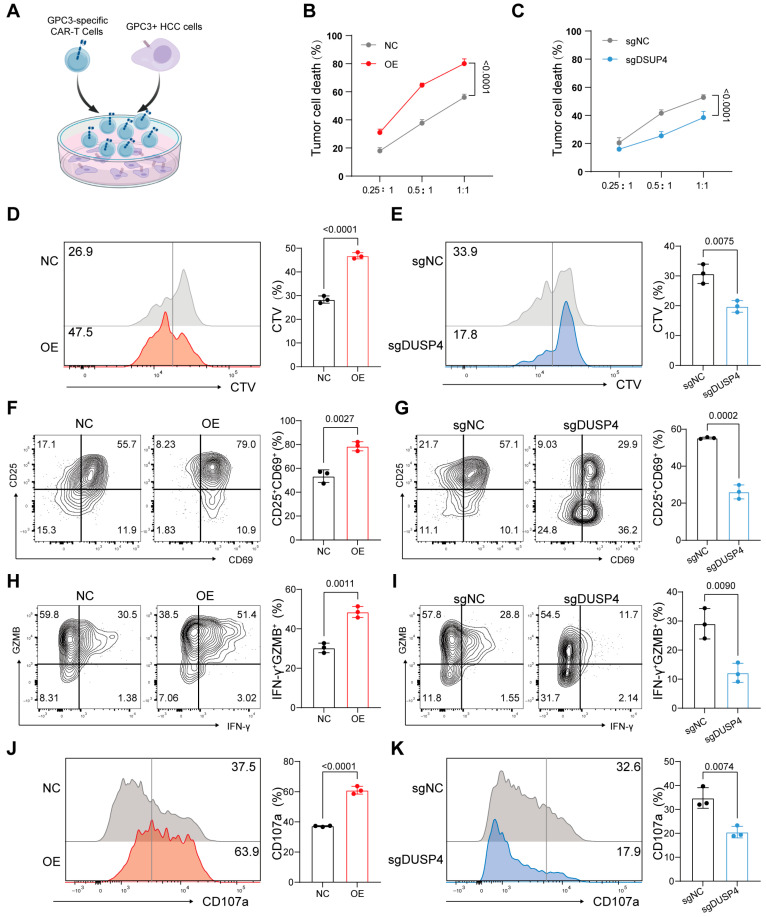
DUSP4 potentiates the killing capacity of T cells in vitro. (**A**) HCC cells were co-cultured with CAR-T cells for co-culture system. Created with BioGDP.com [29]. (**B**,**C**) DUSP4-overexpressing (OE) or DUSP4-knockout (sgDUSP4) Huh-7 cells compared with control cells (NC or sgNC) were separately incubated with T cells at different effector: target ratios. (*n* = 3; presented as means ± SD; two-way ANOVA). (**D**,**E**) OE or sgDUSP4 Huh-7 cells compared with control cells (NC or sgNC) were incubated with Cell Trace Violet (CTV) dye-loaded T cells. Representative histograms of CTV dilution and the calculated proliferation index are shown (*n* = 3; presented as means ± SD; two-tailed unpaired *t*-test). (**F**,**G**) T cells cultured with OE or sgDUSP4 Huh-7 cells assessed by CD25 and CD69 expression, with representative flow plots (**left panel**) and quantification (**right panel**) shown (*n* = 3; presented as means ± SD; two-tailed unpaired *t*-test). (**H**,**I**) T cells cultured with OE or sgDUSP4 Huh-7 cells assessed by IFN-γ and GZMB expression, with representative flow plots (**left panel**) and quantification (**right panel**) shown (*n* = 3; presented as means ± SD; two-tailed unpaired *t*-test). (**J**,**K**) T cells cultured with OE or sgDUSP4 Huh-7 cells and assessed by CD107a expression, with representative flow plots (**left panel**) and quantification (**right panel**) shown (*n* = 3; presented as means ± SD; two-tailed unpaired *t*-test).

Capitalizing on the emergence of CAR-T therapy as a novel cancer treatment modality [26,27] and the established role of GPC3 as an HCC-specific target [28], we investigated whether DUSP4 expression influences tumor cell susceptibility to CAR-T immunotherapy. We performed this assessment by co-culturing GPC3-specific CAR-T cells with the HCC cells, which express the target antigen (Figure 2A). Notably, DUSP4-overexpressing cells exhibited a significant increase in sensitivity to CAR-T-cell-mediated killing compared to control cells (Figure 2B and Figure S2C). Conversely, DUSP4 knockout conferred increased resistance to T-cell-induced killing (Figure 2C and Figure S2D). In co-culture with DUSP4-overexpressing cells, T-cell proliferation was enhanced as measured by CTV dilution assay, while DUSP4-knockout cells produced the opposite effect (Figure 2D,E). Flow cytometric analysis further revealed a marked increase in the proportion of CD25^+^CD69^+^ T cells, which are indicative of early activation, in co-cultures with DUSP4-overexpressing cells and decreased with DUSP4-knockout cells (Figure 2F,G). Moreover, T cells exposed to DUSP4-overexpressing cells showed elevated secretion of the effector molecules IFN-γ and granzyme B (GZMB) (Figure 2H), whereas DUSP4-knockout cells led to reduced secretion of these molecules (Figure 2I). Similarly, surface expression of the degranulation marker CD107a was increased in T cells co-cultured with DUSP4-overexpressing cells and decreased with DUSP4-knockout cells (Figure 2J,K). Collectively, these data demonstrate that elevated DUSP4 expression in HCC cells sensitizes them to T-cell attack in vitro by promoting T-cell expansion, activation, and effector function.

Given that chemotherapy can stimulate anti-tumor immunity and that our prior work showed sorafenib upregulates DUSP4 expression in HCC cells [20], we hypothesized that DUSP4 may mediate chemotherapy-induced immune sensitization. To test this, we pretreated DUSP4-knockdown and control cells with sorafenib prior to co-culture with T cells. Sorafenib pretreatment enhanced T-cell-mediated killing of control cells, but this sensitizing effect was abolished in DUSP4-knockdown cells (Figure S2E), supporting the role of DUSP4 in linking sorafenib treatment to enhanced immune recognition.

### 3.3. DUSP4 Augments T-Cell Activity via Suppression of TGF-β Signaling Pathway

**Figure 3:**
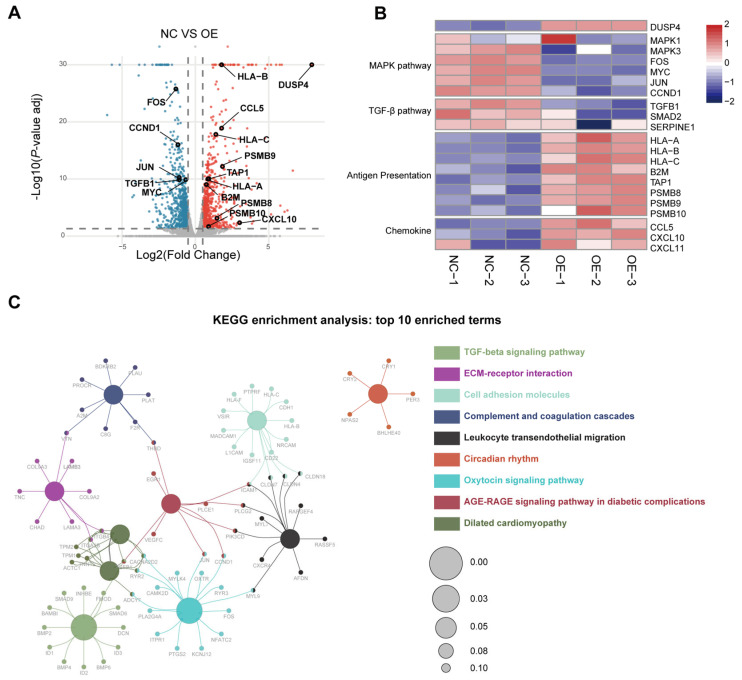
Transcriptomic profiling identifies TGFB1 as a key immunosuppressive cytokine Regulated by DUSP4. (**A**) Volcano plot of differentially expressed genes (DEGs) from RNA-seq analysis in Huh-7 cells: NC versus DUSP4 OE. Red dots, upregulated genes; blue dots, downregulated genes; gray dots, non-significant genes. (**B**) Heatmap showing key genes regulated by DUSP4. (**C**) Top 10 KEGG signaling pathways identified by enrichment analysis using Cytoscape. Pathways and their constituent genes within the same group are coded in identical colors. Shared genes between pathways are connected by lines, and circle size correlates negatively with *p*-value.

To investigate the mechanism through which DUSP4 activates T-cell responses, we performed transcriptomic analysis of DUSP4-overexpressing Huh-7 cells. The results revealed significantly downregulated DEGs encompass components of the MAPK signaling pathway, most notably downstream transcription factors including FOS, MYC, and JUN (Figure 3A,B). Furthermore, DUSP4 overexpression significantly downregulated the immunosuppressive cytokine TGFB1 and markedly upregulated a suite of key antigen-processing (TAP1, PSMB8, PSMB9, PSMB10) and antigen-presentation (HLA-A/B/C, B2M) genes (Figure 3A,B). These alterations likely underpin enhanced tumor cell sensitivity to T-cell-mediated killing. Moreover, RNA-seq analysis revealed increased chemokine expression (Figure 3A,B), which facilitates immune cell infiltration into tumors and thereby potentiates T-cell-mediated killing efficacy. Kyoto Encyclopedia of Genes and Genomes (KEGG) enrichment analysis of DEGs was performed via Cytoscape v3.10.4, identifying the TGF-β signaling pathway as the most significantly enriched pathway (Figure 3C). Similarly, Gene Ontology (GO) analysis identified significant enrichment of terms associated with chemokine and cytokine production (Figure S3A). Collectively, these data support that DUSP4 potentiates T-cell-mediated cytotoxicity toward tumor cells by inhibiting immunosuppressive cytokine production, notably TGF-β1.

Furthermore, the modulation of TGF-β1 expression by DUSP4 at both transcriptional and translational levels was confirmed using qRT-PCR and flow cytometric analysis (Figure S4A–D). ELISA results also confirmed that the concentration of TGF-β1 was significantly diminished in culture supernatants from the DUSP4-overexpressing group and vice versa (Figure S4E–H).

**Figure 4:**
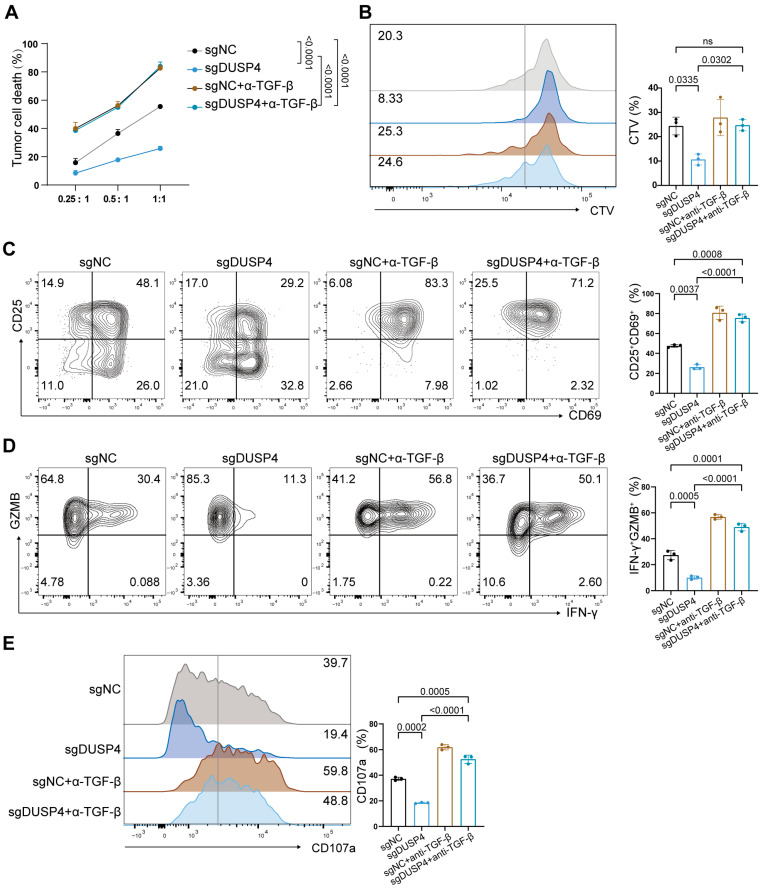
DUSP4 knockout impairs T-cell function in a TGF-β1-dependent manner. (**A**) sgDUSP4 and sgNC Huh-7 cells were separately incubated with T cells at different effector: target ratios. (*n* = 3; presented as means ± SD; two-way ANOVA). (**B**) sgDUSP4 and sgNC Huh-7 cells were incubated with CTV dye-loaded T cells. Representative histograms of CTV dilution and the calculated proliferation index are shown (*n* = 3; presented as means ± SD; two-tailed unpaired *t*-test; ns, not significant). (**C**) T cells cultured with sgDUSP4 or sgNC Huh-7 cells assessed by CD25 and CD69 expression, with representative flow plots (**left panel**) and quantification (**right panel**) shown (*n* = 3; presented as means ± SD; two-tailed unpaired *t*-test). (**D**) T cells cultured with sgDUSP4 or sgNC Huh-7 cells assessed by IFN-γ and GZMB expression, with representative flow plots (**left panel**) and quantification (**right panel**) shown (*n* = 3; presented as means ± SD; two-tailed unpaired *t*-test). (**E**) T cells cultured with sgDUSP4 or sgNC Huh-7 cells and assessed by CD107a expression, with representative flow plots (**left panel**) and quantification (**right panel**) shown (*n* = 3; presented as means ± SD; two-tailed unpaired *t*-test). Cells were treated with 50 ng/mL anti-TGF-β antibody or not.

TGF-β1 is known to inhibit CD8^+^ T-cell function by suppressing the secretion of key effector cytokines such as IFN-γ and GZMB [30], which is consistent with our observation that DUSP4 overexpression in HCC cells reduces cytokine secretion in GPC3-specific CAR-T cells (Figure 2F–J). To investigate whether DUSP4 affects T cells via the TGF-β1 pathway, we blocked the TGF-β1/TGF-βR axis with a neutralizing antibody. In co-culture cytotoxicity assays with GPC3-specific CAR-T cells, DUSP4 knockout decreased T-cell-mediated killing, however, this effect was abolished upon TGF-β1 blockade (Figure 4A). Similarly, the anti-TGF-β antibody mitigated the suppressive impact of DUSP4 knockout on T-cell proliferation (Figure 4B), activation (Figure 4C), cytokine secretion (Figure 4D) and degranulation ((Figure 4E). To further explore the impact of TGF-β1 neutralization on T-cell function under conditions of DUSP4 overexpression, we co-incubated CAR-T cells with tumor cells in vitro. The results demonstrated that TGF-β1 blockade further enhanced T-cell-mediated lysis in the DUSP4-overexpressing group (Figure S5A). However, under co-culture conditions with a high effector-to-target (E: T) ratio, TGF-β1 neutralization had no effect on DUSP4-overexpression-mediated T-cell proliferation or activation levels (Figure S5B,C).

Together, these results demonstrate that DUSP4 knockout in HCC cells inhibits T-cell activity by increasing TGF-β1 secretion, aligning with established reports on the pivotal role of TGF-β1 in regulating the TIME [31].

### 3.4. DUSP4 Remodeling the TIME

**Figure 5:**
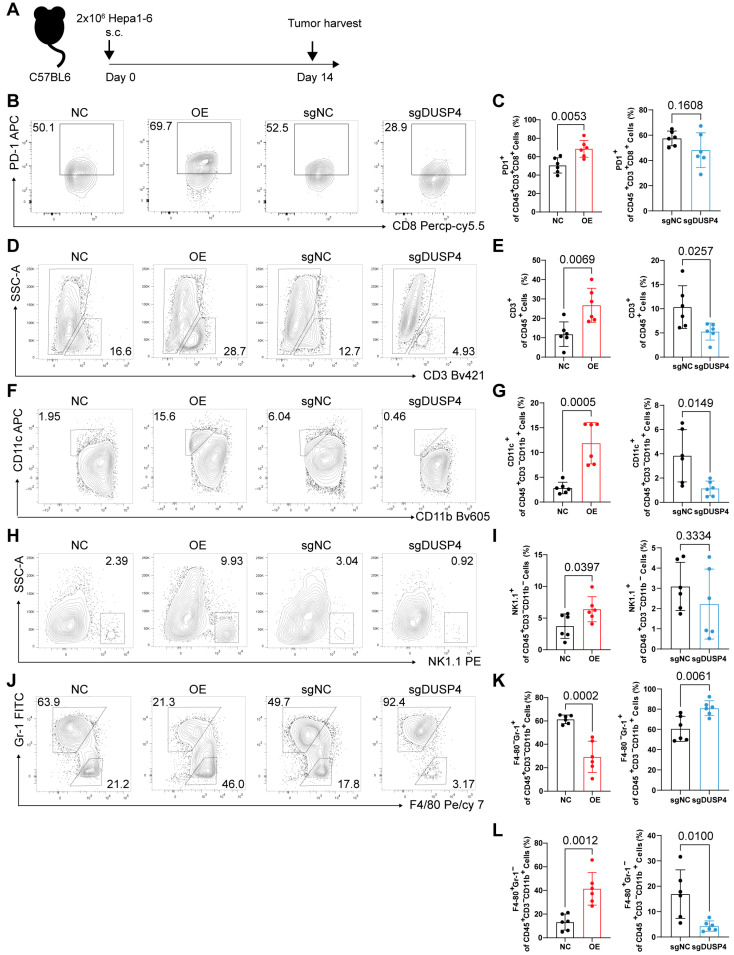
DUSP4-mediated immune microenvironment remodeling in vivo. (**A**) Graphical abstract of the animal study. (**B**) Representative histograms for identifying CD8^+^PD-1^+^ T cells within the tumor microenvironment. (**C**) Quantification of the frequency of tumor-infiltrating CD8^+^PD-1^+^ T cells in OE versus NC group and in sgDUSP4 versus sgNC group. Representative histograms for identifying CD3^+^ T cells (**D**), CD11c^+^CD11b^−^ (DC) cells (**F**), CD11b^−^NK1.1^+^ (NK) cells (**H**), F4/80^low^Gr-1^high^ (MDSC) cells (**J**), F4/80^high^Gr-1^low^ (TAM) cells (**J**), within the tumor microenvironment. Quantification of the frequency of tumor-infiltrating of CD3^+^ T cells (**E**), CD11c^+^CD11b^−^ (DC) cells (**G**), CD11b^−^NK1.1^+^ (NK) cells (**I**), F4/80^low^Gr-1^high^ (MDSC) cells (**K**), F4/80^high^Gr-1^low^ (TAM) cells (**L**) in OE vs. NC group, and in sgDUSP4 vs. sgNC group (*n* = 6; presented as means ± SD; two-tailed unpaired *t*-test).

C57BL/6 mice are one of the most widely used inbred strains in tumor immunology research, characterized by a well-defined genetic background, an intact immune system, and favorable immunocompatibility with syngeneic tumor cells (e.g., Hepa1-6). To evaluate the immune-sensitizing effect of DUSP4 in vivo, we established a subcutaneous tumor model by implanting control, Dusp4-overexpressing, or Dusp4-knockout Hepa1-6 cells into wild-type C57BL/6 mice and subsequently analyzed the TIME by flow cytometry (Figure 5A). Given that PD-1^+^CD8^+^ T cells constitute a critical population enriched for tumor-reactive clones [32,33], we first assessed their infiltration (Figure S6A,B). Dusp4-overexpressing tumors exhibited a significant increase in tumor-infiltrating PD-1^+^CD8^+^ T cells compared to controls (Figure 5B,C and Figure S7A). In addition, CD3^+^ T cells (Figure 5D,E and Figure S7B) and natural killer (NK) cells (identified as CD45^+^ NK1.1^+^) (Figure 5F,G and Figure S6C) were also increased in Dusp4-overexpressing tumors. An increased infiltration of CD11c-positive cells was observed in Dusp4-overexpressing tumors (Figure 5H,I and Figure S7D). These cells predominantly comprise anti-tumor immune subsets, including conventional type 1 dendritic cells (cDC1), whose enrichment is typically associated with favorable patient prognosis and enhanced anti-tumor immunity [34,35]. In contrast, Dusp4-overexpressing tumors showed a marked reduction in key immunosuppressive myeloid populations, including myeloid-derived suppressor cells (MDSCs, identified as CD11b^+^ F4/80^low^Gr-1^high^) (Figure 5J,K and Figure S7E) and tumor-associated macrophages (TAMs, identified as CD11b^+^ F4/80^high^Gr-1^low^) (Figure 5L and Figure S7F).

Collectively, these in vivo data demonstrate that DUSP4 remodels the TIME towards an immunologically active state, characterized by accumulation of cytotoxic lymphocytes and attenuation of myeloid-driven immunosuppression.

### 3.5. DUSP4 Enhances the Efficacy of ICB Therapy

**Figure 6:**
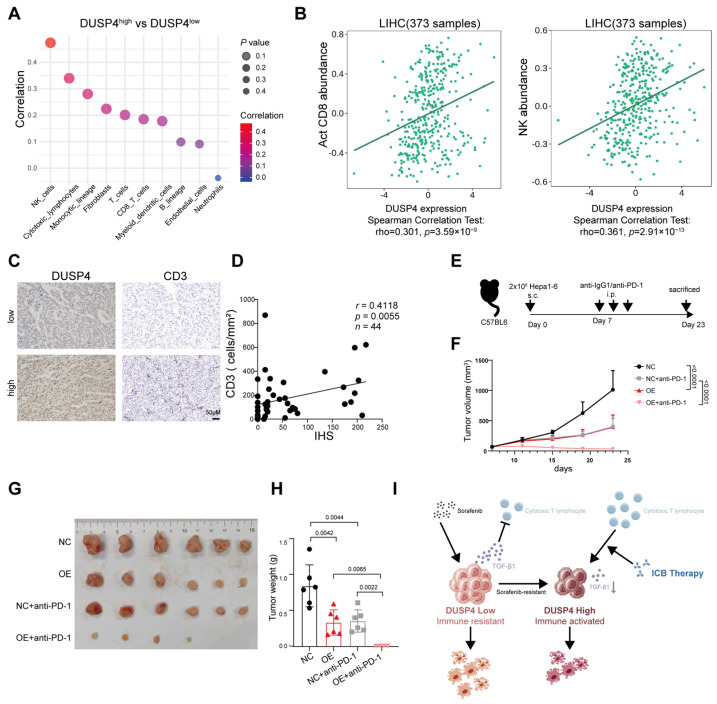
DUSP4-driven T-cell infiltration enhances the response to ICB. (**A**) Association of DUSP4 expression levels (high/low) with immune cell infiltration landscape in TCGA-LIHC cohort, as calculated by the MCPcounter algorithm from the IOBR package (*n* = 363). (**B**) Correlation of DUSP4 expression with activated CD8^+^ T-cell or NK cell abundance in HCC. Data were analyzed using the TISIDB database.Solid lines show the fitted correlations of Act CD8 and NK cell abundances with DUSP4 expression. (**C**) Representative IHC images of DUSP4 and CD3 expression in HCC tissues. Scale bar, 50 μm. (**D**) Analysis of the correlation between DUSP4 immunohistochemical staining intensity and CD3^+^ cell count per unit area (*n* = 44).The line represents the linear trend of the scatter plot. (**E**) Graphical abstract of the animal study. (**F**) Tumor volume in the subcutaneous tumor model (*n* = 6; presented as means ± SD; two-way ANOVA). (**G**) Subcutaneous xenograft tumor model showing the impact of anti-PD-1 treatment in C57BL/6J mice injected with DUSP4-overexpressing cells (*n* = 6). (**H**) Quantification of subcutaneous tumor weights. (**I**) A proposed model illustrating that DUSP4-mediated immunotherapy susceptibility by suppressing TGF-β1 secretion and remodeling the immune-activated microenvironment. Created with BioGDP.com [29].

Analysis of TCGA-LIHC transcriptomic dataset using the MCPcounter algorithm revealed that elevated DUSP4 expression was associated with significantly increased infiltration of effector lymphocytes, including NK cells and T cells, versus DUSP4-low tumors (Figure 6A). Consistent with this finding, analysis of the Tumor-Immune System Interaction Database (TISIDB) further demonstrated a positive correlation between DUSP4 expression and the signature of effector CD8^+^ T cells and NK cells in HCC (Figure 6B). We further validated these computational findings experimentally by performing IHC analysis, which confirmed a notable increase in CD3^+^ T-cell density within tumor tissues exhibiting high DUSP4 expression (Figure 6C,D).

To evaluate the therapeutic potential of DUSP4 modulation in the context of ICB, we established a mouse subcutaneous HCC model. Mice were divided into four groups based on the subcutaneous injection of DUSP4-overexpressing or control Hepa1-6 cells, followed by intraperitoneal administration of an anti-PD-1 antibody (Figure 6E). Compared to control tumors, Dusp4-overexpressing tumors exhibited significantly suppressed growth (Figure 6F). Importantly, administration of the anti-PD-1 antibody more potently attenuated tumorigenesis in mice bearing DUSP4-overexpressing tumors than in controls (Figure 6G,H). These findings suggest that T cells reactivated by α-PD-1 treatment preferentially eliminate HCC cells with elevated DUSP4 expression, a conclusion consistent with the observed enhanced efficacy of α-PD-1 therapy in patients with high DUSP4 expression (Figure 1A,B). Collectively, our data demonstrate that elevated DUSP4 expression sensitizes cancer cells to ICB therapy (Figure 6I).

## 4. Discussion

Precision oncology hinges on the identification of robust biomarkers that can guide therapeutic sequencing and combination strategies. This study elucidates a critical and context-dependent role for DUSP4 in HCC, suggesting it as a potential molecular nexus between sorafenib resistance and immunotherapy response. We demonstrated that sorafenib treatment upregulates DUSP4 expression [20], which not only mediates adaptive resistance to the tyrosine kinase inhibitor sorafenib but also, paradoxically, sensitizes tumors to T-cell-mediated killing and ICB therapy. This apparent functional duality is reconciled by its role as an endogenous regulator of the TIME, primarily through the suppression of immunosuppressive TGF-β signaling. Collectively, our findings suggest DUSP4 as a dynamic biomarker and a novel therapeutic target in the evolving landscape of HCC management.

To facilitate clinical translation, deep-learning-based analysis of IHC and medical imaging represents an emerging and effective approach for biomarker evaluation [36,37]. This strategy could further standardize the assessment of DUSP4 as a candidate biomarker. Specifically, these computational methods enable automated and objective quantification of DUSP4 expression, thereby supporting the development of reliable predictive models and decision-making tools to optimize personalized immunotherapy strategies for HCC.

The success of ICB-based regimens in advanced HCC underscores the therapeutic relevance of the TIME. Herein, we demonstrated that high DUSP4 levels are indicative of an immunologically active TIME and correlate positively with improved response to ICB treatment. Mechanistically, DUSP4 enhanced the susceptibility of HCC cells to T-cell-mediated cytotoxicity, as confirmed in co-culture assays (Figure 2). This pro-immunity role creates an apparent paradox when considered alongside our previous finding that sorafenib upregulates DUSP4 [20]. Therefore, DUSP4 elevation represents an adaptive response that, while protecting tumors from sorafenib, simultaneously sensitizes them to immune attack. This insight reframes sorafenib resistance not merely as a treatment failure but as a potential state of altered immune vulnerability. Consequently, dynamic monitoring of DUSP4 may guide the optimal timing for switching to or combining with immunotherapy.

In HCC, the TGF-β signaling pathway exhibits a well-documented dual phase role, acting as a tumor suppressor in early stages while promoting invasion, metastasis, and immunosuppression in advanced disease [11,13]. This functional switch underscores the necessity of stratifying HCC patients based on TGF-β signaling pathway activity to guide precision therapy. TGF-β and MAPK signaling pathways extensively interact through crosstalk, forming a complex regulatory network [11]. DUSP5 activates the TGF-β/Smad pathway and promotes EMT [38]. Meanwhile, in HCC, DUSP1 modulates TGF-β1 secretion and M2 macrophage polarization through miR-101, thereby influencing the TIME [39]. By contrast, DUSP4 acts as a critical suppressor of TGF-β1-driven oncogenic signaling in several cancer contexts, where its overexpression attenuates TGF-β1-induced ERK activation and inhibits EMT [40]. However, the translational application of strategies targeting the TGF-β1 axis warrants careful consideration of potential off-target effects and systemic toxicities. Systemic inhibition of TGF-β1 risks autoimmune-like toxicities due to its pleiotropic roles in normal tissues [12].

Building on these observations, our study suggests that DUSP4 may function as an endogenous suppressor of TGF-β1-mediated tumor progression in HCC, possibly through dampening MAPK-dependent signaling cascades. Integrating existing literature, we propose a working model: in advanced HCC, loss or low expression of DUSP4 may lead to hyperactive MAPK/ERK signaling, which synergizes with and potentiates the non-canonical (Smad-independent) arms of TGF-β signaling. This enhances TGF-β1 secretion, which in turn dampens tumor antigen presentation and fosters an immunosuppressive TIME, ultimately facilitating immune escape and disease progression. Conversely, high or induced DUSP4 expression, as seen post-sorafenib, dephosphorylates and inhibits key MAPK nodes, thereby attenuating this TGF-β-mediated immunosuppressive program. This shift makes the TIME more permissive for T-cell infiltration and function, explaining the observed ICB sensitization. This model positions DUSP4 as a context-dependent switch whose expression levels help determine whether the TGF-β1 axis exerts predominantly pro-tumorigenic or restrained activity. However, the detailed molecular mechanisms underlying this regulatory axis warrant further investigation.

The immunomodulatory function of DUSP4 is not limited to HCC. A recent study in microsatellite-instable colorectal cancer revealed that DUSP4 acts as a positive regulator of CD8^+^ T-cell infiltration by dephosphorylating CDK7 and promoting CXCL16 expression, while also suppressing ferroptosis in cancer cells [41]. Together with our observations, these results reveal DUSP4 as a conserved immune checkpoint operating across cancer types, wherein distinct effector mechanisms shape the TIME and determine immunotherapy efficacy. We acknowledge that our study focused primarily on the TGF-β1 axis; however, other immune-related pathways are likely involved. Indeed, our RNA-seq data (Figure 3A,B) showed that DUSP4 overexpression also upregulates antigen-presentation genes, suggesting a broader immunomodulatory program beyond TGF-β1 suppression. The findings in colorectal cancer further support the notion that DUSP4 functions through multiple context-dependent mechanisms. Nevertheless, the subcutaneous nature of the mouse model used in this study limits its ability to fully reflect the genuine liver tumor microenvironment. Orthotopic HCC models should be employed in future studies to confirm the functional role of DUSP4 in the native liver context.

Liquid biopsy, TME features, genetic mutations, and multimodal models integrating multiomics data represent promising approaches to guide sequential therapy in HCC [6]. Within the TME, CD8^+^ T-cell infiltration is a key determinant of immunotherapy response, whereas elevated TGF-β1 levels characterize an immunosuppressive TME associated with resistance to ICB [5]. Baseline and on-treatment DUSP4 levels represent candidate biomarkers that may help stratify patients for sequential therapy: sustained DUSP4 elevation during sorafenib treatment could signal both evolving resistance and a potential window of opportunity for subsequent ICB. Furthermore, the regulatory role of the DUSP4/TGF-β1 axis in T-cell function appears generalizable. Our data showed that DUSP4 overexpression enhances the proliferation and cytotoxicity of GPC3-specific CAR-T cells, extending the therapeutic relevance of this axis beyond ICB to ACT and warranting further investigation as a potential target for engineering next-generation immunotherapies.

Looking forward, future efforts will focus on delineating the upstream regulatory networks controlling DUSP4 expression in the TIME and on developing rational combination strategies that leverage DUSP4 modulation to enhance next-generation immunotherapies across solid tumors. However, given that our current findings are primarily derived from GPC3 CAR-T and anti-PD-1 models, it remains unclear whether the DUSP4/TGF-β1 axis similarly modulates responses to other immunotherapeutic modalities, such as bispecific T-cell engagers, CAR-NK cells, or cancer vaccines. Prospective cohort studies and interventional clinical trials are warranted to prospectively validate the clinical utility of DUSP4 in guiding treatment selection and patient stratification. Given the relatively small number of patients available for clinical correlative analysis, especially in the ICB-treated subgroup, our preliminary clinical observations warrant cautious interpretation and require further validation in larger and independent cohorts. Moreover, clinical translation will require rigorous validation of DUSP4 as a dynamic biomarker for treatment selection and the development of pharmacological agents capable of safely restoring or amplifying its activity. Ultimately, integrating DUSP4 status into the molecular stratification of HCC will be instrumental in advancing precision immunotherapy for this aggressive malignancy.

## 5. Conclusions

Our study suggests that DUSP4 may serve as an important regulator of competitive fitness in HCC, shifting its perceived role from a potential marker of targeted therapy resistance to a modulator of the interplay between cancer cells and host immunity. We demonstrate that DUSP4 reprograms the TIME by suppressing TGF-β1 secretion, thereby enhancing T-cell infiltration and activation and converting immunologically “cold” tumors into “hot” ones. Upregulated DUSP4 appears to enhance anti-tumor immunity and increase HCC sensitivity to immunotherapy. These findings position DUSP4 as a promising predictive biomarker for patient stratification and treatment sequencing, as well as a potential therapeutic target for augmenting next-generation immunotherapies across solid tumors.

## Data Availability

RNA-seq data for DUSP4 overexpression in Huh-7 of this study are available at SRA (PRJNA1420459). Other data that were irrelevant for the results presented herein are available from the corresponding authors, HAO, upon reasonable request.

## Supplementary Materials

The following supporting information can be downloaded at: https://www.mdpi.com/article/10.3390/cancers18081289/s1, Figure S1: DUSP4 expression levels stratify immunotherapy response in the GSE235863 cohort; Figure S2: DUSP4 enhances T-cell-mediated cytotoxicity in vitro; Figure S3: GO Clustering analysis of significantly differentially expressed genes in DUSP4-overexpressing cells; Figure S4: DUSP4 modulates TGF-β1 expression in Huh-7 cells; Figure S5: DUSP4 overexpression mimics TGF-β inhibition in promoting T-cell immunity; Figure S6: Hierarchical gating scheme; Figure S7: DUSP4 regulates intratumoral immune cell infiltration; Table S1: List of sgRNA sequences; Table S2: List of antibodies; Table S3: List of primers. File S1: Original images of Western blot in Figure S2A,B.

## Abbreviations

The following abbreviations are used in this manuscript:

HCC	Hepatocellular carcinoma
DUSP4	Dual specificity phosphatase 4
GPC3	Glypican-3
CAR	Chimeric antigen receptor
ICB	Immune checkpoint blockade
TIME	Tumor immune microenvironment
TME	Tumor microenvironment
TGF-β	Transforming growth factor-β
ACT	Adoptive cell therapy
EMT	Epithelial–mesenchymal transition
FBS	Fetal bovine serum
MAPK	Mitogen-activated protein kinase
IPS	Immunophenoscope
LIHC	Liver hepatocellular carcinoma
TCGA	The Cancer Genome Atlas
qRT-PCR	Quantitative real-time polymerase chain reaction
IHC	Immunohistochemistry
PBMC	Peripheral blood mononuclear cell
GZMB	Granzyme B
KEGG	Kyoto Encyclopedia of Genes and Genomes
GO	Gene Ontology
cDC1	Conventional type 1 dendritic cell
MDSC	Myeloid-derived suppressor cell
TAM	Tumor-associated macrophage
TISIDB	Tumor-Immune System Interaction Database
CR	Complete response
PR	Partial response
SD	Stable disease
PD	Progressive disease
OS	Overall survival
PFS	Progression-free survival

## References

[1] Yang X., Yang C., Zhang S., Geng H., Zhu A.X., Bernards R., Qin W., Fan J., Wang C., Gao Q. (2024). Precision treatment in advanced hepatocellular carcinoma. Cancer Cell.

[2] Vogel A., Meyer T., Sapisochin G., Salem R., Saborowski A. (2022). Hepatocellular carcinoma. Lancet.

[3] Singal A.G., Kanwal F., Llovet J.M. (2023). Global trends in hepatocellular carcinoma epidemiology: Implications for screening, prevention and therapy. Nat. Rev. Clin. Oncol..

[4] Kelley R.K., Greten T.F. (2021). Hepatocellular Carcinoma—Origins and Outcomes. N. Engl. J. Med..

[5] Sangro B., Sarobe P., Hervás-Stubbs S., Melero I. (2021). Advances in immunotherapy for hepatocellular carcinoma. Nat. Rev. Gastroenterol. Hepatol..

[6] Yang C., Zhang H., Zhang L., Zhu A.X., Bernards R.A.-O., Qin W.A.-O., Wang C.A.-O. (2023). Evolving therapeutic landscape of advanced hepatocellular carcinoma. Nat. Rev. Gastroenterol. Hepatol..

[7] Sun Q., Hong Z., Zhang C., Wang L., Han Z., Ma D. (2023). Immune checkpoint therapy for solid tumours: Clinical dilemmas and future trends. Signal Transduct. Target. Ther..

[8] Kelley R.K. (2020). Atezolizumab plus Bevacizumab—A Landmark in Liver Cancer. N. Engl. J. Med..

[9] Khanam A., Kottilil S. (2022). New Therapeutics for HCC: Does Tumor Immune Microenvironment Matter?. Int. J. Mol. Sci..

[10] Shimada S., Mogushi K., Akiyama Y., Furuyama T., Watanabe S., Ogura T., Ogawa K., Ono H., Mitsunori Y., Ban D. (2019). Comprehensive molecular and immunological characterization of hepatocellular carcinoma. EBioMedicine.

[11] Xin X., Cheng X., Zeng F., Xu Q., Hou L. (2024). The Role of TGF-β/SMAD Signaling in Hepatocellular Carcinoma: From Mechanism to Therapy and Prognosis. Int. J. Biol. Sci..

[12] Batlle E., Massagué J. (2019). Transforming Growth Factor-β Signaling in Immunity and Cancer. Immunity.

[13] Yang S., Qiu X., Yang Y., Wu J., Wang S., Zheng B., Wu J., Zhou T., Zhang Y., Bai M. (2025). LTA4H improves the tumor microenvironment and prevents HCC progression via targeting the HNRNPA1/LTBP1/TGF-β axis. Cell Rep. Med..

[14] Zhang K., Zhang M., Luo Z., Wen Z., Yan X. (2020). The dichotomous role of TGF-β in controlling liver cancer cell survival and proliferation. J. Genet. Genom. = Yi Chuan Xue Bao.

[15] Zaidi S., Gough N.R., Mishra L. (2022). Mechanisms and clinical significance of TGF-β in hepatocellular cancer progression. Adv. Cancer Res..

[16] Marquardt J.U. (2018). The Role of Transforming Growth Factor-β in Human Hepatocarcinogenesis: Mechanistic and Therapeutic Implications from an Integrative Multiomics Approach. Gastroenterology.

[17] Zandi Z., Kashani B., Alishahi Z., Pourbagheri-Sigaroodi A., Esmaeili F., Ghaffari S.H., Bashash D., Momeny M. (2022). Dual-specificity phosphatases: Therapeutic targets in cancer therapy resistance. J. Cancer Res. Clin. Oncol..

[18] Hu Y., Li Y., Jiang C., Han W., Chen X., Wang P., Xu H. (2025). DUSP9 is Up-Regulated and Promotes Tumor Progression in Head and Neck Squamous Cell Carcinoma. J. Cancer.

[19] Lin H.H., Chang C.W., Liao Y.T., Yeh S.D., Lin H.P., Ho H.M., Cheung C.H., Juan H.F., Chen Y.R., Su Y.W. (2024). DUSP22 inhibits lung tumorigenesis by suppression of EGFR/c-Met signaling. Cell Death Discov..

[20] Hao S.H., Ma X.D., Xu L., Xie J.D., Feng Z.H., Chen J.W., Chen R.X., Wang F.W., Tang Y.H., Xie D. (2024). Dual specific phosphatase 4 suppresses ferroptosis and enhances sorafenib resistance in hepatocellular carcinoma. Drug Resist. Updat. Rev. Comment. Antimicrob. Anticancer Chemother..

[21] Jiang P., Gu S., Pan D., Fu J., Sahu A., Hu X., Li Z., Traugh N., Bu X., Li B. (2018). Signatures of T cell dysfunction and exclusion predict cancer immunotherapy response. Nat. Med..

[22] Yoshihara K., Shahmoradgoli M., Martínez E., Vegesna R., Kim H., Torres-Garcia W., Treviño V., Shen H., Laird P.W., Levine D.A. (2013). Inferring tumour purity and stromal and immune cell admixture from expression data. Nat. Commun..

[23] Charoentong P., Finotello F., Angelova M., Mayer C., Efremova M., Rieder D., Hackl H., Trajanoski Z. (2017). Pan-cancer Immunogenomic Analyses Reveal Genotype-Immunophenotype Relationships and Predictors of Response to Checkpoint Blockade. Cell Rep..

[24] Becht E., Giraldo N.A., Lacroix L., Buttard B., Elarouci N., Petitprez F., Selves J., Laurent-Puig P., Sautès-Fridman C., Fridman W.H. (2016). Estimating the population abundance of tissue-infiltrating immune and stromal cell populations using gene expression. Genome Biol..

[25] Li B., Li Y., Zhou H., Xu Y., Cao Y., Cheng C., Peng J., Li H., Zhang L., Su K. (2024). Multiomics identifies metabolic subtypes based on fatty acid degradation allocating personalized treatment in hepatocellular carcinoma. Hepatology.

[26] Liu X., Liu Y., Zhao D., Shan D., Guo C., Jia L. (2025). Nanomaterials Mediated Enhancement of CAR-T for HCC: Revolutionizing Immunotherapy Strategies. Int. J. Nanomed..

[27] Steffin D., Ghatwai N., Montalbano A., Rathi P., Courtney A.N., Arnett A.B., Fleurence J., Sweidan R., Wang T., Zhang H. (2025). Interleukin-15-armoured GPC3 CAR T cells for patients with solid cancers. Nature.

[28] Haruyama Y., Kataoka H. (2016). Glypican-3 is a prognostic factor and an immunotherapeutic target in hepatocellular carcinoma. World J. Gastroenterol..

[29] Jiang S., Li H., Zhang L., Mu W., Zhang Y., Chen T., Wu J., Tang H., Zheng S., Liu Y. (2025). Generic Diagramming Platform (GDP): A comprehensive database of high-quality biomedical graphics. Nucleic Acids Res..

[30] Nixon B.G., Gao S., Wang X., Li M.O. (2023). TGFβ control of immune responses in cancer: A holistic immuno-oncology perspective. Nat. Rev. Immunol..

[31] Yin C., Zhang C., Wang Y., Liu G., Wang N., Liang N., Zhang L., Tu Q., Lv J., Jiang H. (2025). ALDOB/KAT2A interactions epigenetically modulate TGF-β expression and T cell functions in hepatocellular carcinogenesis. Hepatology.

[32] Ye J., Cao W., Tao Z., Zhao S., Wang C., Xu X., Zhao A., Gao J. (2021). Novel method for effectively amplifying human peripheral blood T cells in vitro. Exp. Cell Res..

[33] Liu Y., Lu P., Ma Y., Yin Z., Xu H., Xiang L., Zhang W., Li S., Liang X. (2022). Peripheral Polyfunctional PD1(+) CD8(+) T cells demonstrated strong immune protection in non-small cell lung cancer. Eur. J. Immunol..

[34] Morita S., Lei P.J., Shigeta K., Ando T., Kobayashi T., Kikuchi H., Matsui A., Huang P., Pittet M.J., Duda D.G. (2025). Combination CXCR4 and PD-1 Blockade Enhances Intratumoral Dendritic Cell Activation and Immune Responses Against Hepatocellular Carcinoma. Cancer Immunol. Res..

[35] Ma H., Kang Z., Foo T.K., Shen Z., Xia B. (2023). Disrupted BRCA1-PALB2 interaction induces tumor immunosuppression and T-lymphocyte infiltration in HCC through cGAS-STING pathway. Hepatology.

[36] Kumar N., Akram W., Bhushan M., Lakhnotra A., Manhas J. (2025). Empirical Analysis of the Performance of Machine Learning Algorithms in Classifying 2D MR Images from PCA Reduced HOG and LBP Features. Biomed. Inform. Smart Healthc..

[37] Gupta R.K., Kaur M., Manhas J. (2019). Tissue Level Based Deep Learning Framework for Early Detection of Dysplasia in Oral Squamous Epithelium. J. Multimed. Inf. Syst..

[38] Fan W., Xing Y., Yan S., Liu W., Ning J., Tian F., Wang X., Zhan Y., Luo L., Cao M. (2024). DUSP5 regulated by YTHDF1-mediated m6A modification promotes epithelial-mesenchymal transition and EGFR-TKI resistance via the TGF-β/Smad signaling pathway in lung adenocarcinoma. Cancer Cell Int..

[39] Wei X., Tang C., Lu X., Liu R., Zhou M., He D., Zheng D., Sun C., Wu Z. (2015). MiR-101 targets DUSP1 to regulate the TGF-β secretion in sorafenib inhibits macrophage-induced growth of hepatocarcinoma. Oncotarget.

[40] Guler S., Altunok T.H., Sarioglu A., Zik B., Asmaz D., Kayapunar N., Sonmez O., Tepedelen B.E., Yalcin A. (2022). Overexpression of dual-specificity phosphatases 4 and 13 attenuates transforming growth factor β1-induced migration and drug resistance in A549 cells in vitro. Biochem. Biophys. Res. Commun..

[41] Zhang D., Yang S., Xu H., Chen Z., Wang X., Sun Y. (2025). The novel role of DUSP4 in suppressing ferroptosis and promoting cytotoxicity of CD8(+) T cells in MSI colorectal cancer. Br. J. Cancer.

