# Supplementary material for: Dual Specificity Phosphatase 4 Enhances Immunotherapy Response by Inhibiting TGF-β1 Secretion in Hepatocellular Carcinoma
Source: Cancers (Basel). 2026 Apr 19;18(8):1289. doi: 10.3390/cancers18081289 (PMC13115269; doi:10.3390/cancers18081289)
Supplement: Supplementary file 1 [file cancers-18-01289-s001.zip › Supplementary Material.pdf]

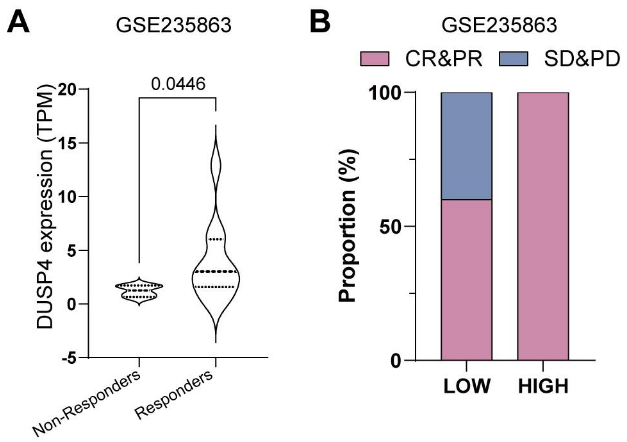

Figure S1. DUSP4 expression levels stratify immunotherapy response in the GSE235863 cohort. (**A**) Violin Plot showing the difference in DUSP4 expression levels between the non-responder and responder group. (**B**) Bar chart showing the DUSP4 expression levels in CR/PR vs SD/PD.

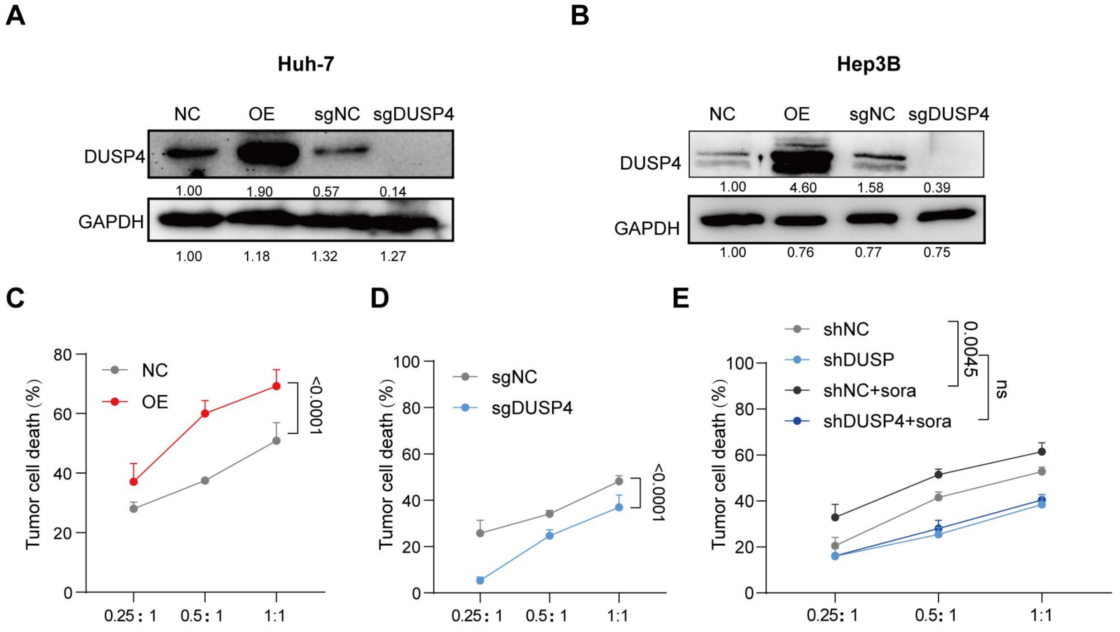

Figure S2. DUSP4 enhances T-cell-mediated cytotoxicity *in vitro*. **(A-B)**. WB results showing the protein expression of DUSP4 in Huh-7 cells and Hep3B cells. **(C)**. T cell-mediated tumor cell-killing assay using OE or NC cells. **(D)**. T cell-mediated tumor cell-killing assay using sgDUSP4 or sgNC cells (n = 3; presented as means ± SD; two-tailed unpaired t test). **(E)**. Cytotoxicity assay of T cells against tumor cells following 24-hour sorafenib (10μM) pretreatment (n = 3; presented as means ± SD; two-tailed unpaired t test). Original western blots are presented in Figure S8.

A

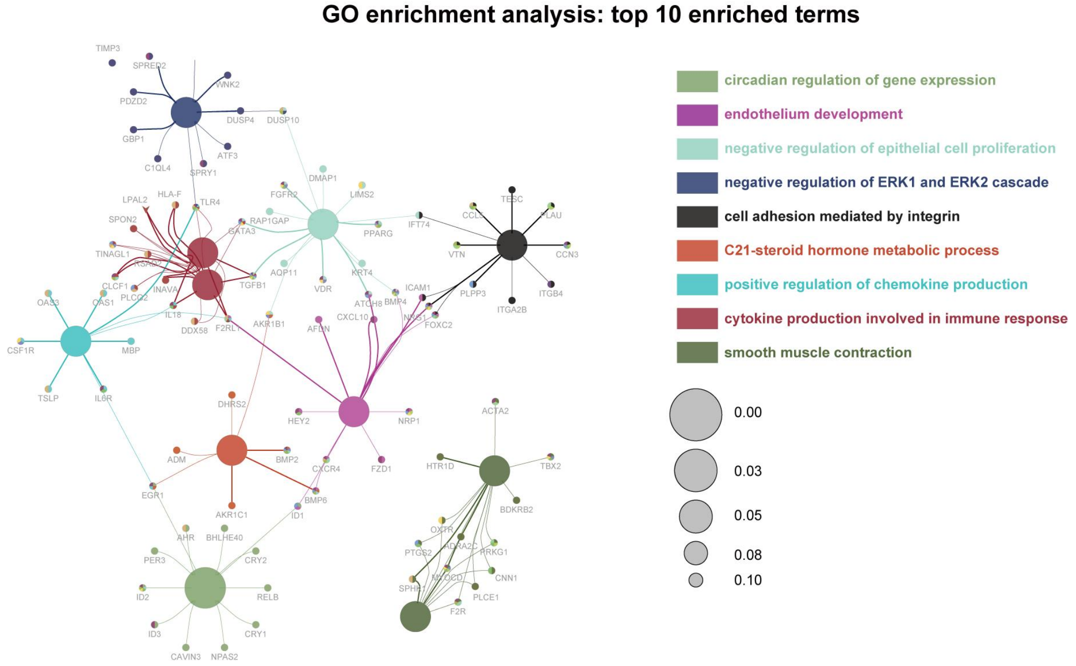

Figure S3. GO clustering analysis of significantly differentially expressed genes in DUSP4-overexpressing cells. **(A)**. Top 10 GO terms identified by enrichment analysis using Cytoscape. Pathways and their constituent genes within the same group are coded in identical colors; shared genes between pathways are connected by lines, and circle size correlates negatively with *P*-value.

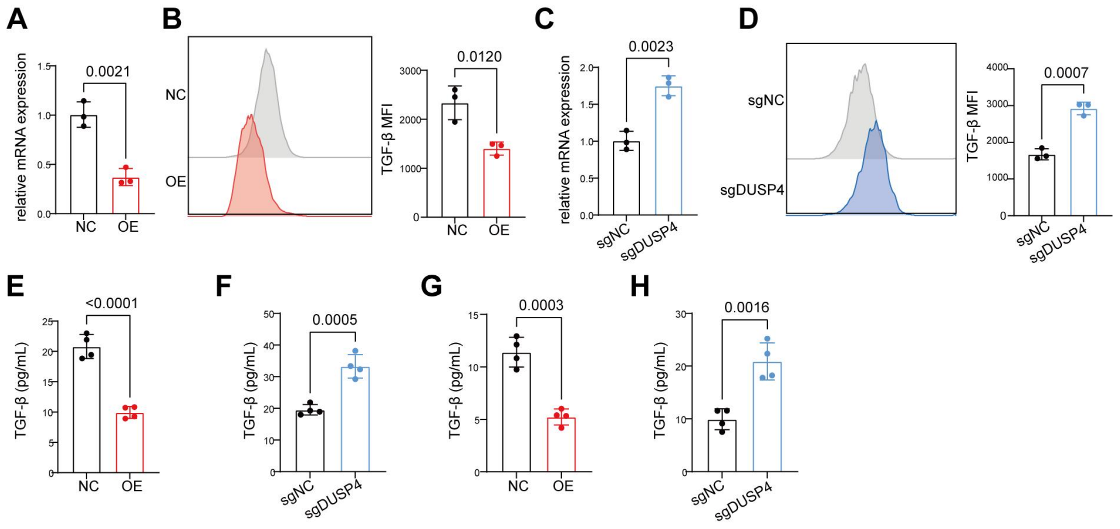

Figure S4. DUSP4 modulates TGF-β1 expression in Huh-7 cells. **(A-B)**. The mRNA expression level of TGF-β1 was measured by qRT-PCR in OE and NC Huh-7 cells (n = 3; presented as means ± SD; two-tailed unpaired t test). **(C-D)**. Flow cytometric analysis of intracellular TGF-β1 expression in sgNC and sgDUSP4 Huh-7 cells (n = 3; presented as means ± SD; two-tailed unpaired t test). **(E-F)**. ELISA for secreted TGF-β1 in supernatants of Huh-7 with DUSP4 overexpression or knockout (n = 4; presented as means ± SD; two-tailed unpaired t test). **(G-H)**. ELISA for secreted TGF-β1 in supernatants of Hep3B with DUSP4 overexpression or knockout (n = 4; presented as means ± SD; two-tailed unpaired t test).

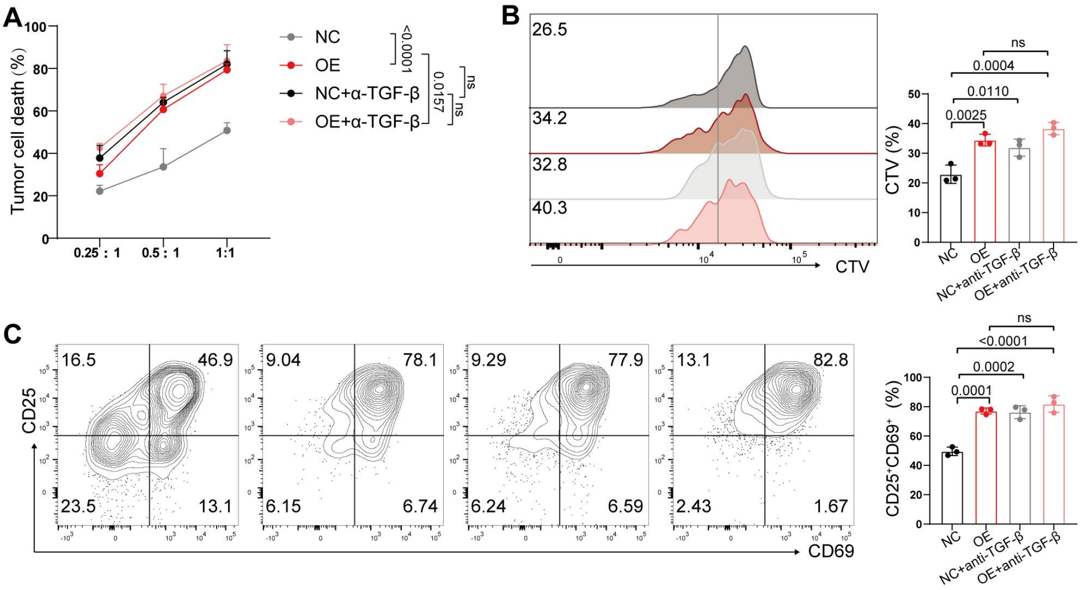

Figure S5. DUSP4 overexpression mimics TGF-β inhibition in promoting T cell immunity. (**A**). DUSP4-overexpressing and NC Huh-7 cells were separately incubated with T cells at different effector: target ratios (*n* = 3; presented as means ± SD; two-way ANOVA). (**B**). DUSP4-overexpressing and NC Huh-7 cells were incubated with CTV dye-loaded T cells. Representative histograms of CTV dilution and the calculated proliferation index are shown (*n* = 3; presented as means ± SD; two-tailed unpaired *t* test) (**C**). T cells cultured with DUSP4-overexpressing and NC Huh-7 cells assessed by CD25 and CD69 expression, with representative flow plots (left panel) and quantification (right panel) shown (*n* = 3; presented as means ± SD; two-tailed unpaired *t* test).

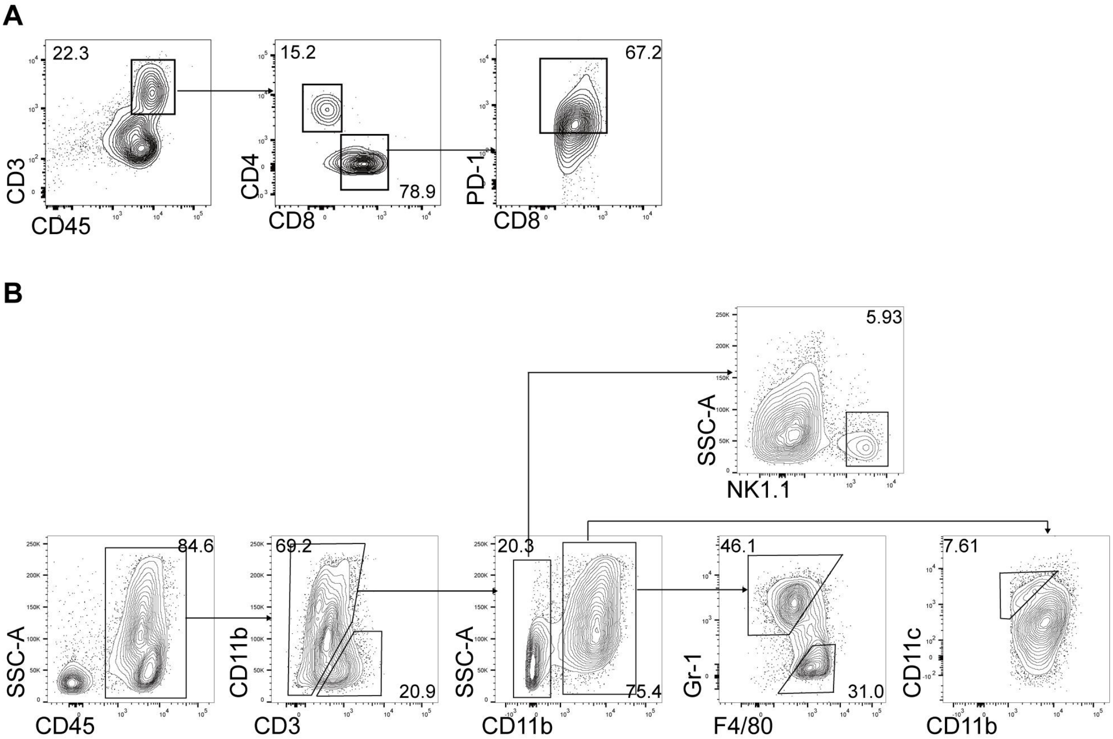

Figure S6. Hierarchical gating scheme. **(A)**. Gating strategy for identifying tumor-infiltrating $CD8^{+}PD1^{+}$ T cells. **(B)**. Gating strategy for identifying tumor-infiltrating myeloid cell subsets.

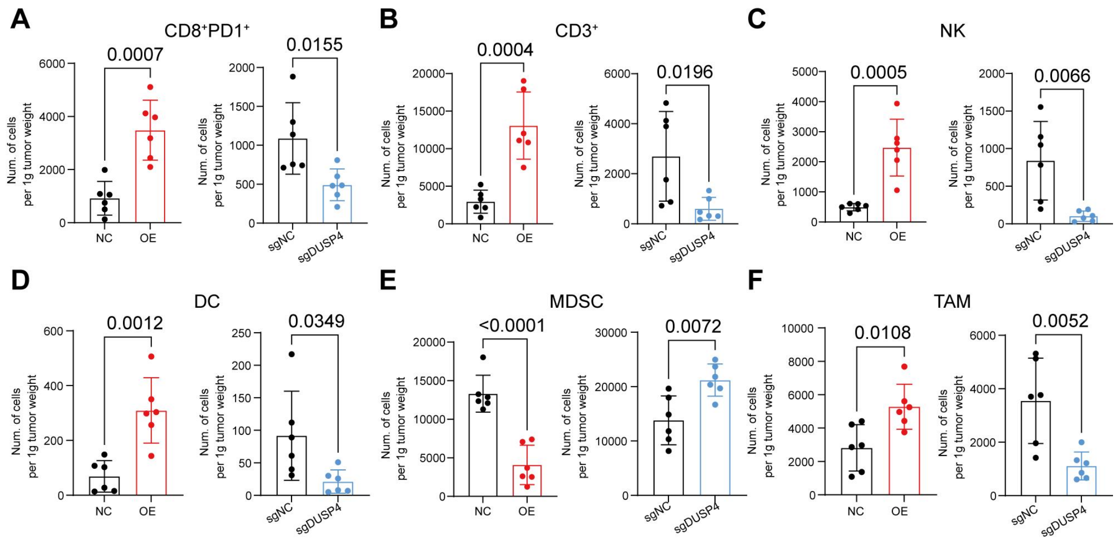

Figure S7. DUSP4 regulates intratumoral immune cell infiltration. **(A)**. Absolute numbers of $CD8^+PD\text{-}1^+$ T cells per gram of tumor tissue. (**B-F**). Absolute numbers of $CD3^+$ T cells**(B)**, $CD11c^+CD11b$- (DC) cells**(C)**, CD11b-NK1.1$^+$(NK) cells**(D)**, F4/80^{low}Gr-1^{high} (MDSC) cells**(E)**, F4/80^{high}Gr-1^{low} (TAM) cells**(F)** per gram of tumor tissue (n = 6; presented as means ± SD; two-tailed unpaired t test).

Table S1. List of sgRNA sequences

Name	Sequence (5'-3')
sgCTRL	GTTCCGCGTTACATAACTTA
DUSP4-sgRNA-1	AGGTTCGGTCAACGTGCGCT
DUSP4-sgRNA-2	TCAGTACAAGTGCATCCCAG

Table S2. List of antibodies

Name	Manufacturer	Lot. No.	Application	RRID
Anti-DUSP4	ProMab	P07284	WB	AB_3738380
Anti-GAPDH	Proteintech	60004-1-Ig	WB	AB_2107436
PE/cy7 anti-mouse CD25	BioLegend	102016	FC	AB_312865
Bv605 anti-mouse CD4	BioLegend	100547	FC	AB_11125962
Bv605 anti-mouse CD11b	BioLegend	101237	FC	AB_11126744
AF488 anti-mouse FOXP3	BioLegend	320012	FC	AB_439748
PE/cy7 anti-mouse F4/80	BioLegend	123114	FC	AB_893478
APC/cy7 anti-mouse CD45	BioLegend	103116	FC	AB_312981
Bv421 anti-mouse CD3	BioLegend	100227	FC	AB_10900227
PerCP anti-mouse CD8	BioLegend	100732	FC	AB_893423
PE anti-mouse IFNγ	BioLegend	505808	FC	AB_315402
APC anti-mouse Granzyme B	BioLegend	372204	FC	AB_2687028
APC anti-mouse CD11c	BioLegend	117310	FC	AB_313779
FITC anti-mouse Gr-1	BioLegend	108406	FC	AB_313371
FITC anti-human CD25	BioLegend	985812	FC	AB_2922662
APC anti-human CD69	BioLegend	310910	FC	AB_314845
PE anti-human CD8	BioLegend	980902	FC	AB_2616623
PB anti-human IFNγ	BioLegend	502522	FC	AB_893525
FITC anti-human GZMB	BioLegend	515403	FC	AB_2114575
Bv605 anti-human CD4	BioLegend	300556	FC	AB_2564391
APC/cy7 anti-human CD3	BioLegend	300318	FC	AB_314054
Anti-CD3	Zhongshan Jinqiao Biotechnology	ZM-0417	IHC	AB_2890105

Table S3. List of primers

Primer name	qPCR primer sequence (5’→3’)
hDUSP4-Forward	GGCATCACGGCTCTGTTGAAT
hDUSP4-Reverse	GTCGGCCTTGTGGTTATCTTC
hTGFB-Forward	GGCCAGATCCTGTCCAAGC
hTGFB-Reverse	GTGGGTTTCCACCATTAGCAC
hGAPDH-Forward	TGCACCACCAACTGCTTAGC
hGAPDH-Reverse	GGCATGGACTGTGGTCATGAG
mDUSP4-Forward	GTACATCGACGCAGTAAAGGAC
mDUSP4-Reverse	GCTTGACGAACTCAAAAGCCTC
mGAPDH-Forward	AGGTCGGTGTGAACGGATTTG
mGAPDH-Reverse	GGGGTCGTTGATGGCAACA
