# Supplementary figures and images for: Dual Specificity Phosphatase 4 Enhances Immunotherapy Response by Inhibiting TGF-β1 Secretion in Hepatocellular Carcinoma
Source: Cancers (Basel). 2026 Apr 19;18(8):1289. doi: 10.3390/cancers18081289 (PMC13115269; doi:10.3390/cancers18081289)

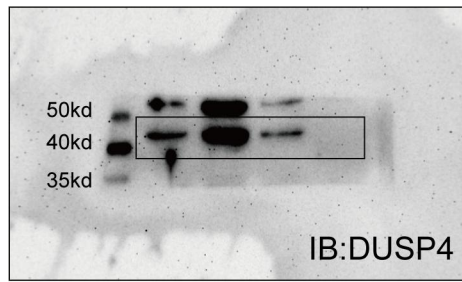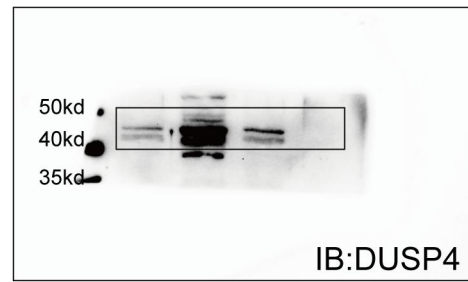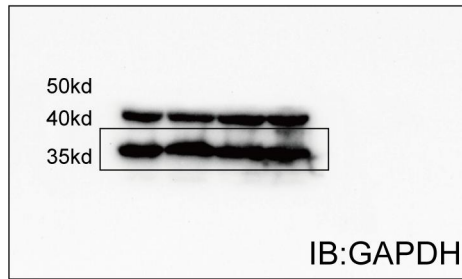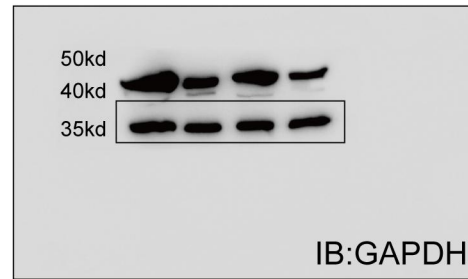

File S1. Original images of western blot in Figure S1A and S1B.

Supplement: Supplementary file 1 [file cancers-18-01289-s001.zip › File S1. Original western blots.pdf]
